# Supplementary material for: Are carbon emissions trading and green financial instruments synergistic? -Comprehensive quantitative research based on content analysis
Source: PLoS One. 2024 Mar 7;19(3):e0298601. doi: 10.1371/journal.pone.0298601 (PMC10919601; doi:10.1371/journal.pone.0298601)
Supplement: S1 Data — (ZIP) [file pone.0298601.s001.zip › Data/地级市Z_F工作报告.zip!/─■╧─/╥°┤¿╩╨/└┤╫╘╣1⁄2╓┌║┼í╛┬φ┐╦╩2╛▌═°í┐_2017.docx]

各位代表：

现在，我代表市人民政府向大会报告工作，请予审议，并请市政协委员和其他列席人员提出意见。

一、四年来的主要工作

市十四届人大一次会议以来的四年，是银川实现发展理念重大转变、发展内涵显著提升、发展动力持续增强的四年，是经受各种困难和挑战，逆势而上、顺势而为，取得改革发展新成就的四年。在自治区党委、政府和市委的坚强领导下，市政府团结带领全市人民，认真落实“2258”工作思路，加快推进开放内涵式发展，干在实处、走在前列，全市经济保持稳步快速增长。经济总量连续跨越1200亿元至1500亿元四个台阶,预计年底达到1580亿元，是2012年的1.4倍(下同)，年均增长9%，占全区半壁江山；规上工业增加值525亿元,年均增长10.1%，工业增加值跃居西北省会第二；固定资产投资1695亿元，年均增长16.5%；社会消费品零售总额513亿元，增长1.6倍，年均增长9.2%；公共财政预算收入188亿元，增长1.66倍，年均增长13.6%；城镇和农村居民人均可支配收入达到30552元和12096元，年均增长8.8%和9.7%；一二三产结构调整为3.9:51.3:44.8，三产比重提升3个百分点。

四年来，我们树立世界眼光，强化战略思维，推进开放内涵式发展，银川在全国的战略地位明显跃升。主动承担为国家和自治区向西开放闯新路、做贡献的重要任务；国务院批复《银川城市总体规划》,“西北地区重要的中心城市”定位得到强化，辐射带动功能不断增强，居住在银川、创业在银川等七大品牌进一步凸显。承担着行政审批改革、公立医院综合改革等十多项重要改革的试点任务，是国家全面深化改革“试验田”之一。举办承办的WCA世界电竞大赛、TMF全球智慧城市峰会和亚洲都市景观奖颁奖礼成为四大世界级盛会品牌。银川从不沿边、不靠海的西部小省会，跃升为内陆开放的前沿阵地；从国家能源和优势农产品的“大后方”跃升为东西双向开放的重要承接地。

四年来，我们认识和引领新常态，优化布局，调转结合，积极应对经济下行。不断强化对新常态下速度换档、结构调整、动力转换的规律性认识，适时调整出台工业19条、房地产16条等150余项政策措施，综合运用财政、税收、金融等调控工具，对冲经济下行，实现了从资源型向多元型经济的转变，从土地财政向新经济财政的转变，从传统旧动能向质量和效益并重的新动能转变，经济呈现出较强“抗压性”。一是着力优化发展布局应对经济下行。在区域布局上，强化“全域银川”理念，推进永贺灵与中心城区一体化进程，开辟滨河新区功能区，城市由“阅海时代”向“黄河时代”跨越，由单核突进向双核驱动转变，城市规划面积扩展到275平方公里，建成区面积增加了34平方公里。在产业布局上，“稳一、优二、提三、育新”，规上工业中“两化融合”企业占66.5%，优势主导产业向集群化方向发展；实施“反梯度”发展战略，大数据、电子竞技等新产业新业态率先布局、蓬勃发展。在园区布局上，新建综合保税区、通航产业园和临港经济区，IBI和TMT育成中心融合发展，银川经济技术开发区等8大园区对GDP的贡献率达到40%，成为引领全区转型升级的领头羊。二是依靠科技创新引领工业转型。建成国家科技成果转移转化平台３个，省部级共建实验室20家、工程技术研究中心30家，发展高新企业51家，研发经费投入年均增长25%，为经济转型“加钢淬火”。铸件3D打印、羊绒分梳、煤基烯烃等一批关键核心技术全球领先。现代纺织、先进装备制造等优势特色产业集群占规上工业增加值的88%。以舍弗勒轴承、小巨人机床、巨能机器人、银川大河数控等为代表的先进装备制造业占据国际高端市场，智能制造水平居西北第二；以天通、佳晶蓝宝石和隆基硅为代表的新材料、新能源产业成为世界单打冠军；以伊品、泰瑞为代表的生物医药加速向“微笑曲线”两端攀升；以中银、恒天如意为代表的现代纺织崛起，带动轻工业上调6.5个百分点。三是着力培育新产业新业态促进转型。加强与中兴通讯合作，建成大数据中心一期，引进“好大夫”“微医”等大数据企业13家。荣获“中国数字化转型领军用户奖”。WCA电竞大赛成为世界最大的第三方电竞平台；建成银川通航机场暨通航产业园；培育“双创”基地（空间）100个，共享装备进入国家首批“双创”示范基地，众创、众包、众筹、众扶四众拾柴添薪助力。新经济发展一枝独秀，带动税收增长12.8%，高于传统产业7.5个百分点。四是加快发展现代服务业带动转型。服务业增加值年均增长8%。大阅城、新华联、万达广场、国际会议中心、润恒城、公铁物流中心等一批“双十工程”和物流园区建成运营，现代物流总额达到2660亿元。以信息产业、知识产权转化为核心的生产性服务业加速发展，IBI育成中心实现主营业务收入60亿元。实施军博园、三沙源等一批重大文化旅游项目，旅游收入达到110亿元，入选十大国家旅游休闲示范城市。会展业荣获国家“金海豚”大奖。五是大力发展“三精”农业带动转型。粮食生产“十三连丰”，建成国家级现代农业示范基地27个，培育农业龙头企业223家，农产品加工转化率达到64%,贺兰山东麓葡萄酒、灵武长红枣等一批名优特农产品出口30多个国家和地区。六是优化投资结构引领转型。持续开展“准备之冬、建设之春、发展之夏、收获之秋”活动，实施项目4306个，三产投资占比达到60.8%，提高9.7个百分点。创新投融资模式，运用共享思维聚合资源、资本，打造智慧城市、恒天如意纺织产业等一批标杆。

四年来，我们着力打造“碧水蓝天明媚银川”的城市名片，推动“美丽银川”向“美好银川”转变。加快完善基础设施配套，建成三座黄河大桥，打通新建扩建主干道路13条200公里，改造小街巷55条15公里，“八横十八纵”路网构建起城市“半小时”经济圈。公共交通机动化出行分担率提高5.4个百分点。获批第二批全国地下综合管廊试点城市。实施旧城（棚户区）改造，拆迁1661万平米，新建安置区30个570万平米，20万人乔迁新居。实施既有节能改造830万平米。新建15个小城镇、49个中心村，水电路气网等基础配套七到位。被列为第三批国家新型城镇化综合试点城市。坚持城市精细化管理和农村环境综合整治并举，“数字城管”“以克论净”等新模式推广应用。全国城乡环境卫生整洁行动现场会在永宁县召开。坚持生态建设和污染防治并举，实施110国道、滨河新区等大整治大绿化工程，新建续建徕龙、唐徕等市民休闲公园39个、小微公园60个，绿博园建成投用。治理沙化和荒漠化土地180万亩，城市绿地率、绿化覆盖率和人均公园绿地面积分别达到40.99%、41.03%和16.57平米，居全国前列。实施西部水资源综合利用及艾依河、宝湖等扩整和岸线景观提升工程，新增水域1.4万亩，湖泊湿地面积占建成区的十分之一。实施“蓝天工程”，拆除燃煤小锅炉548台，淘汰黄标（老旧）车4.3万辆，启动华电灵武电厂“东热西送”工程。“塞上湖城”“碧水蓝天、明媚银川”成为城市靓丽名片。开展“书香银川、银川书香”等活动，评选“最美银川人”571名。入选国家园林城市和健康城市，荣获亚洲都市景观奖和中国人居环境奖。

四年来，我们不断深化改革，打破体制坚冰，发展活力进一步迸发。一是供给侧改出新“蓝海”。去产能，淘汰钢铁加工、金属冶炼等落后产能158.16万吨；去库存，制定并落实货币化安置、农民工及乡村教师购房等政策，去化周期控制在合理范围；降成本，累计减少企业用电、税收等成本16亿元；补短板，银西高铁动工兴建，包银高铁获批实施，百姓期盼多年的高铁梦即将变为现实；青银高速、乌玛高速改扩建完工，火车站综合枢纽等重大项目加快实施。二是“放管服”改革走在全国前列。推行“一枚印章管审批，一个大厅办成事、一个平台全监管，一个号码优服务”审批服务模式，企业和群众办事效率提升86%；新增市场主体13.29万户，年均增长27%，民间投资增速居全国前列。三是金融创新实现重大突破。开展“四个清理”，盘活国有资产302亿元；搭建金融控股、西部担保等金融创新体系，设立产业基金21支，组建通联资本、新兴产业资本等国有资本运营公司12家，吸引撬动社会资本600亿元助力产业转型升级；36家企业在“新三板”挂牌；金融占服务业比重由17%提升到28%。四是农村改革顺利推进。完成农村土地承包经营权确权登记142万亩。开展农村集体经济组织股权制度改革试点，实施“双增双减”“双富”工程，102个空壳村全部脱壳。五是社会事业改革持续深化。“先住院后付费”“先诊疗后付费”模式在全国推广。文化体制改革实现了公共服务与文化产业发展的双赢。教育改革实现投资和办学体制的多元化，进入全国中小学教育质量综合改革试验区。社会保障经办实现“五险合一”。

四年来，我们搭建平台，连接通道，对外开放迈出扎实步伐。主动融入国家“一带一路”发展倡议，加快建设“三位一体”开放核心区。滨河新区累计投资320亿元，实施基础设施和重大产业项目150个，“四城”建设强力推进。综合保税区获批肉类（水果、种苗）进境口岸，成功加入世界自由区组织。阅海湾中央商务区“三园一轴”及宁夏国际会堂等成为对外开放新地标，28家总部企业、419家金融企业入驻。打通陆路、空中、网上“丝绸之路”，实现货物出口交货值87.92亿元。建成河东机场三期，开通11条国际航线、70条国内航线，年吞吐旅客突破630万人次；与丝路沿线10个关区实现通关一体化，开通国际货运班列。引进500强企业24家，完成招商引资到位资金2339.3亿元，年均增长13.74%，外贸进出口总额年均增长9.5%。

四年来，我们不断加大投入，完善机制，人民生活质量稳步提升。公共财政的80%以上用于改善民生。兑现为民承诺实事47件。推进创业带动就业，高校毕业生当年就业率保持在90%以上，城镇年均新增就业6.15万人，累计转移农村劳动力就业40万人次，被国务院授予“全国创业先进城市”。加快完善社会保障体系。实施被征地农民养老保险，惠及7.47万人。首推大病医保上不封顶和特困家庭救助制度，基础养老金标准居西部省会首位；建设各类保障房240万平米，圆了7万多低收入群体安居梦。投资34.5亿元，建成半子沟等移民安置区26个，精准扶贫减少深度贫困人口7.24万人，移民人均可支配收入年均增长33%以上，闽宁协作模式全国推广。投入100亿元建成中小学、幼儿园200余所，率先在全区实现义务教育基本均衡全覆盖。实施国际医疗城、口腔医院等一批健康医疗项目，卡瓦心脏中心、韩国JK美容整形等国际高端医疗服务资源落户银川。建成韩美林艺术馆、银川当代美术馆和华夏河图艺术家村等一批重大文化项目，镇村（居）文化设施实现全覆盖，成功举办全国文化馆年会等重大节会。持续推进社会治理创新，交通、文化和体育等7项基本公共服务满意度进入全国38个主要城市前十，智慧城市建设提高了百姓的幸福指数，荣获“全国综合治理先进市”。依法妥善处置“1·05”公交纵火案。精准防控贺兰山“8·21”特大洪灾，实现零伤亡。安全生产形势稳定向好，事故起数下降41%。荣获“全国双拥模范城”八连冠和全国人民防空先进城市。民族团结、宗教和顺成为另一张靓丽名片。人才、工会、共青团、妇联、残联、文联、科协、防震减灾、气象、档案、史志、红十字会等工作都取得了新成绩。

四年来，我们坚持为民务实清廉，深入推进职能和作风转变，政府自身建设进一步加强。自觉接受人大依法监督、政协民主监督和社会监督，认真办理区市人大议案建议、政协提案2327件。健全完善法治政府建设指标体系和政府法律顾问制度。制定《智慧城市建设促进条例》等地方性法规22部、政府规章9部，每年依法公开信息8万余条。荣获“中国政府创新优秀实践奖”“中国法治政府奖提名奖”。完成新一轮政府机构改革，设立行政审批服务局、金融工作局、人才工作局和大数据管理服务局。编制政府权力清单、责任清单、市场准入负面清单，精简行政职权65.4%，行政职权在西北省会中最少。严格执行中央“八项规定”、国务院“约法三章”和区市若干规定，国家土地、审计、环保督查和自治区巡视问题整改率达到98%。制定行政问责制度13项，创新开展“电视问政、电视施政”，剑指“四风”及不作为、慢作为等突出问题，先后问责224个单位2300余人。“三公”经费大幅压减，公车改革走在西部前列。

各位代表，过去四年，从发展实践看，我们经受住了转型升级初期最严峻的考验，有效应对了经济下行，保持了一定的发展速度；从城市综合竞争力看，银川在国家大格局中的位置发生了积极变化，城市综合竞争力指数排名前移；从老百姓的收获看，我们不断补齐社会民生短板，人民群众有了更多获得感。四年的成绩来之不易，四年的体会与经验弥足珍贵，需要在今后工作中牢牢汲取，不断发扬光大。

一是必须坚持以新发展理念为指导，实施“反梯度”发展战略。理念决定思路、思路决定出路。我们牢固树立“五大发展理念”，适应和引领发展新常态这个经济工作大逻辑，在主动服务国家战略中，深刻认识和精准把握市情，认真落实“双调双转”和“2258”工作思路和任务，实施“反梯度”发展战略，一步接轨产业前沿、零度承接产业高端，新技术、新产业、新业态、新模式加速成长催生经济增长新动能。

二是必须坚持以改革创新为引领，推进开放内涵式发展。改革创新是推动发展的动力和源泉。我们始终立足银川欠发达的实际，牢牢抓住发展这个第一要务不放松，用好国家赋予宁夏内陆开放型经济试验区先行先试政策，主动融入国家“一带一路”倡议，通过一系列创新举措，探索出了一条内陆欠发达地区开放内涵式发展的有效路径。

三是必须坚持以问题为导向，锐意进取、攻坚克难。坚持问题导向是治国理政的基本工作方法。我们直面经济社会发展重大难题和事关群众切身利益的突出问题，一件一件去研究解决和落实，逢山开路、遇河架桥，通过开辟滨河新区功能区、创新智慧城市模式等，破解了发展空间不足、发展新动能不足和城市病等瓶颈，在解决问题中突破，在突破中实现发展。

四是必须坚持以为民谋福祉为中心，切实办好民生实事。民生连着民心，民心关系国运。我们坚持把维护和发展好群众根本利益放在第一位，提出以干部的工作高度提升银川的发展高度，以干部的辛苦指数换取群众的幸福指数，不断加大民生投入，切实办好棚户区改造、道路畅通等为民实事，赢得了群众的信任和支持，全市上下凝聚起了共同干事创业的强大合力。

五是必须坚持以“功成不必在我”的境界，敢于担当、主动作为。一代人有一代人的使命，一代人有一代人的担当。我们深刻认识到银川事业是历届班子工作的传承、延续和发展，既要一任接着一任干、一张蓝图绘到底，也要立足当前、着眼未来，干好打基础利长远的事。只要以敢为人先的胆略和运筹得当的智慧，抓铁有痕、踏石留印，久久为功，就一定能够带领全市干部群众克服一切困难，创造无愧于时代、无愧于人民的辉煌业绩。

这些经验是发展实践告诉我们的，也是市十四次党代会要求我们传承和坚持的，经验十分宝贵，成绩鼓舞人心，是自治区党委和市委坚强领导以及人大、政协关心支持的结果，是全市上下同心同德奋力拼搏的结果。在这里，我代表市人民政府，向全市人民，向给予政府工作大力支持的人大代表、政协委员，向各民主党派、工商联、各人民团体和社会各界人士，向驻银中央和自治区各单位，向驻银解放军、武警消防官兵、公安干警，向关心和支持银川发展的社会各界朋友们，表示崇高的敬意和衷心的感谢！

各位代表，成绩属于过去，未来仍需努力。在银川发展新的长征路上，我们还存在一些突出问题、面临不少挑战。一是发展不足、规模较小、质量不高是我们最大的实际，尤其是与西部发展较快的城市相比还有不少的差距，亟待在新一轮发展中实现赶超。二是转型升级任务艰巨，传统产业转升步子不大，新产业新业态新经济刚刚起步，缺技术、缺人才、缺资金等发展瓶颈需持续破解。三是投资强度放缓、驱动能力减弱，缺乏一批支撑力强、关联度高的大项目、好项目。四是增收压力依然较大，受经济下行和结构性减税等因素影响，财政、居民持续增收困难加大，非税收入占比较高，刚性支出不断扩大，收支矛盾加剧。五是开放水平还不高，平台、通道和辐射源（点）建设还处在培育阶段，与内陆开放型经济试验区核心区不相匹配。六是政府职能转变还不到位，干部队伍中还存在一定的松懈情绪、不善作为等现象，责任担当和依法履职能力迫需改进。对此，我们一定要高度重视，采取有效措施，认真加以解决。

二、今后五年的工作思路和目标任务

本届政府任期内是率先建成全面小康社会的决胜阶段，也是创新发展、转型追赶的关键时期。宏伟的事业需要精准的定位和方向指引。今年,习近平总书记、李克强总理亲临宁夏·银川视察指导，对行政审批改革、智慧城市、闽宁扶贫协作等工作给予高度评价。特别是总书记亲自主持召开了载入史册的“银川会议”，给我们鼓劲加压、把脉导航，作出了“贯彻新的发展理念，主动服务和融入国家发展战略，立足自身优势，努力实现经济繁荣、民族团结、环境优美、人民富裕，确保与全国同步建成全面小康”的重要指示，为银川的发展确立了新坐标、明确了新定位、赋予了新使命。自治区要求银川继续走在前列、作出表率。市第十四次党代会提出了“践行新理念、发展新经济、实现新跨越，为早日全面建成小康社会而奋斗”的目标。我们要强化首府使命和责任担当，遵循“社会主义是干出来的”质朴真理，弘扬“不到长城非好汉”的宁夏精神，主动作为、创新实干。

政府工作总体要求是：高举中国特色社会主义伟大旗帜，以邓小平理论、“三个代表”重要思想、科学发展观为指导，深入贯彻落实党的十八大以来中央各项决策部署、习近平总书记系列重要讲话精神，以“四个全面”战略布局统领各项工作，深入践行“五大发展理念”，按照自治区第十一次党代会以来各项部署及市第十四次党代会精神，解放思想、真抓实干，全面推进开放内涵式发展，努力建设“两宜两化”和西北地区重要的中心城市，早日全面建成小康社会，谱写好伟大中国梦的银川篇章。

各位代表，科学把握发展大势，方能紧跟时代潮流。当前，银川正处于重大历史机遇最集中的阶段，处于转型升级、新旧动能转换最紧要的当口，改革发展不进则退。纵观国际风云变幻，不确定、不稳定因素增多，新一轮科技革命、产业变革正在迭加释放发展新机遇、新空间，尤其是在“互联网+”时代，产业要向三维智能科技型转化，企业公司组织要向平台化方向演进，这种发展趋势不可逆转，发展势头不可阻挡。我国发展面临经济下行和转型升级双重压力，但经济潜力足、韧性强、回旋余地大，长期向好的基本面没有变，正在孕育着拥抱新经济体系的重大发展机遇；特别是随着国家“一带一路”倡议的实施和宁夏内陆开放型经济试验区的建设，先行先试政策正逐步呈现溢出效应，为我市创新发展、转型追赶带来巨大的势能。立足当下看银川，历届班子打下的坚实基础，正在催生新的发展力量。新时代，新机遇，新期待。今后五年，我们将深入贯彻落实“五大发展理念”，以推进供给侧结构性改革为主线，努力把银川开放内涵式发展推向新阶段，实现经济繁荣、民族团结、环境优美、人民富裕。

——“经济繁荣”，就是引领经济发展新常态，传统产业转型升级迈向中高端，新经济新产业成为经济发展的主动力。综合实力大幅提升，位次在全国省会中前移。地区生产总值年均增长8%左右；固定资产投资年均增长10%左右；规上工业增加值年均增长8.5%以上；公共财政收入年均增长7.5%以上。

——“民族团结”，就是共同团结奋斗、共同繁荣发展的主题得到深化，各民族更加和睦相处、融合共生。用团结推动发展，用发展促进团结，民族事务法制化水平显著提升，民族团结的基础更加牢固，社会主义新型民族关系更加和谐，成功创建全国民族团结进步模范市。

——“环境优美”，就是舒朗、大气、清爽的城市风格更加清晰、特色更加鲜明，现代化城市功能和品质更加完善，空气质量优良天数达到80%以上，黑臭水体全面消除，工业自备井全部关停，饮用水水源地和重要水功能区水质达标率达到100%，“碧水蓝天、明媚银川”的城市品牌更加靓丽，实现“六城联创”目标。

——“人民富裕”，就是实现全面小康的短板逐步消除，基本公共服务均等化和社会保障水平走在全国前列。社会消费品零售总额年均增长8%；城镇和农村居民人均可支配收入年均增长8%和8.5%；公民具备科学素质比例达到10%以上，人均预期寿命达到78岁以上，率先在自治区和西北省会城市建成全面小康社会。

（一）建平台、聚人才、强投入，建设“创新型银川”。主动融入全球科技革命浪潮，统筹理念创新、科技创新、产业创新、制度创新，建成国家“创新型城市”。优化完善创新体系，挖掘用好“3+1”动力，完善平台搭建、奖励补助、成果转化应用等全链条扶持机制，推进科技创新与金融试点城市建设，利用多层次资本市场，促进创新链、产业链、资金链、政策链紧密衔接，到2021年全社会研发投入占GDP比重达到3.5%，科技进步贡献率达到55%以上。搭建功能完备的科技创新系列平台，支持清华、浙大等知名院校搭建产业技术研究院和创新联盟，力争建设2家以上国家级研发平台，新认定10家以上区级工程技术研究中心，培育一批科技型企业。坚定不移走银川特色的自主创新之路，力争在能源化工、智能制造、现代纺织和新材料等领域掌握一批世界前沿的核心技术。加快建设人才特区，着眼产业转型升级，引进一批具有国际水准的创新型科技领军人才、专家学者和创新团队，培育一批实用型技术人才。争取建设博士后研究站、博士后创新示范中心。完善对科研人员的股权、期权及分配激励机制，让做出突出贡献的人“名利双收”，让世界级人才拿到世界级待遇。努力营造创新文化氛围，着力构建集研发、孵化、融资、服务和园区承接、知识产权保护等于一体的创新生态系统。

（二）调结构、强动能、促转型，打造银川经济升级版。主动融入国际产业分工，推动一二三产融合发展，加快构筑以新兴产业为先导、高端服务业为主体、新型工业和现代都市农业为基础的现代产业体系。着力实施“兴工强市”战略。落实“中国制造2025”，实施绿色制造工程，促进制造业研发、设计、生产、市场经营全链条信息化改造，鼓励企业建立智能化工厂、数字化车间和柔性制造单元，提高信息化水平；支持共享铸钢、巨能机器人等50家示范企业依靠互联网汇聚全球智慧，形成新的产业动能。大力实施“千百亿”工程。继续做大能源化工、现代纺织两个千亿级产业集群，推进煤化工、化纤、纺织、制衣和时尚品牌关联技术互通互融；推动先进装备制造、生物医药、新能源、新材料等10个百亿级产业集群化发展。力争到2021年规模以上工业增加值突破800亿元。着力实施新产业新业态“反梯度”发展战略。对标国际产业前沿、瞄准产业高端，促进通航、电商、电竞、新能源汽车、大健康等新产业加速布局发展，催生一批附加值高、竞争力强的新的经济增长点。着力抓好现代服务业发展。加快建设国家现代服务业综合试点城市。推动现代商贸服务、文化创意、第三方物流、融资租赁、服务外包、信息技术等生产性服务业向专业化和价值链高端延伸；推动现代物流、全域旅游、健康养老、餐饮住宿等生活性服务业向精细化和高品质转变。着力抓好“三精农业”。加快构建现代农业产业体系，大力发展“1+2+4”优势特色产业，力争占比达到90%以上；加快建立现代农业生产体系，标准化生产和农产品质量安全追溯体系全覆盖；加快建立现代农业经营体系，适度规模经营和主要农产品加工转化率均达到70%以上。创建第三批国家食品安全示范城市。

（三）搭云桥、建云链、释云能，建设新型智慧城市。加快“宽带中国”示范城市建设，以“互联网+”带动新兴产业发展、传统产业改造，通过“+互联网”为城市治理、民生改善插上互联网翅膀。大力发展信息经济。完善产业扶持政策，加快智慧银川大数据二期、智慧城市产业园等重点项目建设，再吸引一批新业态落户银川；搭建国家级云平台、数据库，培育发展基于互联网的个性化定制、众包设计、云计算、数据服务等新型产业模式，衍生一批智慧产业和传统产业融合的应用链、创新链、发展链，打造西北信息经济发展高地。持续推进信息惠民。着眼于城市治理现代化、社会服务精细化、市民生活便捷化，拓展智慧交通、智慧旅游、智慧医疗、智慧教育等服务领域，收集开放涵盖信用、科技、环境、安监、气象等300多项数据，再研究开发一批智能化应用项目，力争在社会服务领域全覆盖、有提高。

（四）抓统筹、强基础、补短板，加快建设“银川都市区”。主动担当西北重要中心城市责任，统筹推进区域空间布局、城市功能提升、城乡一体化发展。推进全域空间一体化。认真落实银川空间发展战略规划（多规合一），适时修编城市总体规划，实现一本规划、一张蓝图、一套体系管全域。加快推进永宁、贺兰撤县设市（区），与主城区一体化发展，推动灵武与宁东、滨河新区、综保区、临港经济区融合互动，申创国家级新区。按照舒朗、大气、清爽的城市风格和不同的功能定位，支持兴庆区开展城市空间修复，打造特色街区，改造老旧小区，增加公共绿地。支持金凤区优化行政服务空间布局，建设阅海经济区，打造总部经济中心、金融服务中心。支持西夏区实施“西夏突破”，实现由工业主导型向现代服务业，尤其是旅游业转型，建设一批特色鲜明的镇、街（村）、院（点）。推进全域重大基础设施一体化。坚持适度超前和适度举债原则，争取国家批复建设地铁1号和2号线，力争建成旅游环线轻轨、银川至宁东轻轨和银新铁路有轨电车、永贺线城市轨道交通、银川至巴彦浩特快速铁路、银西高铁、火车站综合客运枢纽、大团结广场客运枢纽等重大基础设施项目，力争列入全国综合客运枢纽示范城市。推进全域城乡发展一体化。按照农村人口向城镇中心村集中，就业创业人口向功能区集中的基本原则，补齐城乡基础设施和社会配套服务短板，全面消除城中村、城市棚户区，农村人均公共服务配套占有率达到西北最高，城市公共服务满意度进入全国主要城市前五。打造一批特色小镇，抓好空间布局，明确产业定位，坚持市场主体，强化项目组合，使系列特色小镇成为新型产业的聚集地、创客发展的承载地、宜居宜游的乐园。推进全域生态建设一体化。实施大绿化、城市景观提升、湖泊水系连通和城市防洪工程，严守生态红线保护好城市稻田，打造黄河两岸绿色化、湖泊湿地景观化、贺兰山东麓田园化和城区花园化的自然生态景致，建设“湖泊城市”“田园城市”“森林城市”“花园城市”，到2021年，建成区绿化覆盖率达到43%，绿地率达到42%，森林覆盖率达到18%。

（五）强平台、筑通道、稳支点，打造内陆开放新高地。主动融入中蒙俄、新亚欧大陆桥、中巴等六大经济走廊，实施“1431”开放战略，推进基础设施和产业互联互通，打造丝绸之路经济带重要节点城市。做强开放平台。滨河新区要对标国际产业合作园区，完善服务配套、创新管理机制，承接国际国内健康、医疗、科技、教育等产业转移。综合保税区主动对接国内外自贸区，加强与乌力吉、霍尔果斯、青岛等口岸的联动发展，争取国家批复药品、整车进口等更多指定口岸，积极申建国家自贸区。力争进出口贸易总额和利用外资年均增长10%。提升陆路空中网上通道开放水平。加快完善现代化立体交通体系，启动建设银川机场四期，开通更多国际国内航班和旅游班列，争取将银川建设成中东、西亚、中亚国家与国内其他城市联通的重要空中枢纽。打造多侧供给的开放支点。举办承办好TMF全球智慧城市峰会、WCA世界电竞大赛、亚洲都市景观奖颁奖礼和国际马拉松比赛等国际性重大节会赛事。鼓励有实力的企业走出国门收购国外品牌和核心技术，开展兼并重组。争取在新能源汽车、太阳能、风能、金融、文化传播等领域开展合作。以更为务实的合作成效，为国家与海合会自贸区谈判并签署自贸协定创造有利条件，做出银川担当。

（六）兜底线、织密网、惠民生，建设“和谐银川”。坚持以发展红利和改革红利不断满足人民群众对美好生活的向往。提高城乡居民收入水平。实施收入倍增计划，增加居民经营性、财产性收入，到2020年城乡居民收入比2010年翻一番。完善落实就业创业政策，研究制定小微双创指数评价体系和科技型小微企业认定标准，争创西北“中国青创汇”创业创新平台，打造西部“双创”高地。提高脱贫质量。落实东西部扶贫协作座谈会精神，深入实施产业扶贫、教育扶贫、金融扶贫、健康扶贫四大工程，建立精准扶贫、稳定脱贫长效机制，全面打赢脱贫攻坚战。提高社会保障能力。建成更高标准、更加公平、更可持续的社会保障体系。促进公共服务均等化。学前教育实现全覆盖、义务教育实现优质均衡发展，建设具有国际水平的区域职业教育高地。基本形成分级诊疗格局，实现互联网医疗和家庭签约医生广覆盖，人人享有均等化的基本医疗和基本公共卫生服务。养老护理、康复、保健等大健康产业蓬勃发展，“健康银川”成为城市品牌。公共文化服务体系更加完善，基本形成10分钟便民文化圈和运动休闲健身圈，建成具有重要影响力的区域文化中心和国家文化消费试点城市。提高社会综合治理能力。坚持以安全发展为首要，及时消除各个领域安全隐患，公共安全、社会治理能力进一步提升，公众安全感进一步提高。全面推开“互联网+”工作模式，依靠信息技术，建成更高水平的“平安银川”。争创国家社会信用体系建设示范城市、“全国双拥模范城”九连冠。巩固深化全国文明城市创建成果，培育和践行社会主义核心价值观，用中国梦凝聚共识、汇聚力量，逐步形成银川城市精神内核和城市品格，推动美丽银川加速向美好银川转变。

三、2017年主要工作

今年是新一届政府的开局之年，也是供给侧结构性改革的深化之年。我们要认真贯彻落实中央经济工作会议、农村工作会议，以及自治区党委十一届九次全会、市委十四届二次全会等重要会议精神，坚持稳中求进总基调，适应把握引领经济发展新常态，全力做好稳增长、促改革、调结构、惠民生、保稳定等各项工作。今年主要预期目标：地区生产总值增长8%左右，公共财政预算收入增长7.5%左右，全社会固定资产投资增长10%左右，社会消费品零售总额增长8%，外贸进出口总额增长8.5%左右，城镇和农村居民可支配收入分别增长8%和8.5%左右，在实际工作中争取更好结果。完成自治区下达的主要污染物控制性指标。

（一）打好项目投资攻坚战。坚持“经济工作项目化”，实施913个项目，年度计划投资2240亿元。狠抓重大项目带动。积极抓好银西高铁、包银高铁、银川至宁东轻轨、京藏高速改扩建等区级重大项目建设。全力抓好100个市级重点项目，完成投资380亿元。抓好黄河航运一期，沈阳路、宝湖路向西延伸，永宁黄河大桥东连接线、西延伸段，10条40公里城市地下综合管廊等重大基础设施建设项目。加快推进共享铸造智能工厂、西轴轨道交通轴承、隆基硅3GW单晶硅棒和切片、智慧城市产业园等重大产业项目建设。强化项目要素保障。完善落实项目包抓等推进服务机制，倒排工期、挂图作战、奖优罚劣；制定投资核准事项清单，推行工程建设项目多评合一、多审合一、多图合一、联合验收等新模式。继续做大产业基金、西部担保规模，鼓励企业对接资本市场，增强股权、债券和票据等直接融资能力，争取200亿元国家各类资金（债券），多措并举解决项目融资难题。开展大招商大引资活动。加快推进招商引资从单一引进向组团式引进转变，从注重资金引进向注重模式引进转变，从政策优惠引进向环境优化引进转变。认真落实“银川会议”精神，抓住全国工商联常委会在银召开等重大机遇，策划举办工商联会员企业、民营500强企业进银川等系列活动;区市联动，创新与东部地区产业合作模式，共建飞地产业园;引进世界和中国500强企业3家，力争6月底完成1200亿元招商引资任务。

（二）推进产业提升“四大工程”。深化产业成龙配套，完善链条，增强竞争力。抓好工业转型升级工程。实施180个工业项目，规模以上工业增加值增长8.5%。现代纺织产业集群，加快推进纺织园“一城三园”建设，实施恒天如意1亿米衬衫面料和1000万米精纺呢绒面料、年产16万吨涡流纺精梳纱线等项目。先进装备制造产业集群，抓好恒天国基机器人产业园、力成智能数字化工厂、利勃海尔智能化工厂等项目，推进铸造3D打印、高端精密轴承等智能制造产业化，争创国家智能制造示范区。新能源新材料产业集群，开工建设金龙、卡威新能源汽车项目，启动实施恒天碳纤维产业基地项目，支持隆基硅、天通和佳晶蓝宝石扩大产能、延伸产业链，从单打冠军向行业集团军升级。生物产业集群，加快推进伊品清洁化生产、紫荆花秸秆造纸、国际医疗城、宁医大总院生物科技信息产业园等项目，推动生物产业向生命科学领域延伸。抓好新产业新业态培育壮大工程。大数据产业集群，完善产业发展规划，加快实施智慧产业大厦等项目，支持“返空汇”、景联科技等互联网企业发展，成立大数据产业联盟。电竞产业集群，规划建设世界电竞大厦、世界网游开发创业园，吸引国际电竞、网游高端企业集聚，办好WCA常态化赛事。通航产业集群，实施通航小镇、通航博物馆、西部通航飞机制造项目，组建通航俱乐部，推动飞机制造组装、航空运动及飞行体验、航空物流产业布局发展，办好第三届西部航展。抓好农业提质增效工程。实施现代园区提升、农业品牌创优等“六大工程”。抓好贺兰山东麓葡萄文化长廊和阿瓦隆郁金香种植园、康乃馨工厂化基地建设，新增鲜花基地8000亩、酿酒葡萄4000亩。推进沿山沿河两大乡村旅游带建设，支持四十里店村、原隆村建成特色产业村。加快推进农业科技创新和推广应用，大力发展农村电商，鼓励农民创业，促进农村一二三产业融合发展。建设高标准农田45万亩。抓好服务业优化提升工程。出台支持现代物流业发展政策，试点开展物流实名制和物流行业电子票据化。加快推进西夏工人文化宫、永泰城等项目建设运营，支持公铁物流中心开展商业模式创新。高标准办好国际产业合作峰会、电商博览会等46场次展会。加快建设国家电子商务示范城市，完善电子交易、物流、结算三大服务平台，支持贺兰县、灵武市建设全国电子商务进农村综合示范县。支持IBI与TMT育成中心发展以信息技术应用为核心的高端生产服务业。统筹全域旅游“一轴两带”布局，编制五年发展规划，加快推进三沙源等项目建设，新建华强方特主题文化公园、亚高原青少年足球训练基地。抓好贺兰山岩画等历史遗迹保护开发，争创2家国家5A级旅游景区。办好国际岩画艺术节、全国青少年阳光体育大会等活动赛事。

（三）推进“美丽银川”建设“三大行动”。全面提升“两宜两化”城市建设水平。开展完善城乡基础配套行动。加强大团结广场、览山公园东侧等重点区域的城市设计。改造长水巷、金城北巷等10条小街巷，改造民族花园、幸福苑等54个老旧小区86.8万平米。打造“公交都市”，启动建设西夏广场等综合枢纽及公交首末站，实施银新铁路有轨电车、109国道东移（兴庆段）、文昌街南延伸等道路畅通工程，新建停车场10处，利用小街巷增加停车位。新开辟优化调整公交线路24条。抓好闽宁、镇北堡等6个美丽小城镇及杨家寨、五渡桥等28个“美丽村庄”建设。开展生态绿化品质提升行动。实施大环境绿化、园林景观提升等53个生态建设项目，新建水洞沟生态公园、10个小微公园和一批儿童主题公园，建成国际鲜花港、花博园水街等项目，举全市之力办好第九届中国花博会、国际荷花节。推进生态绿化管护机制改革，成立园林绿化国有公司，探索政府购买服务、PPP合作等模式，实现“建管分离、效酬兼济”。开展“蓝天碧水·绿色城乡”专项行动。抓好中央环保督察问题整改落实工作。加快华电灵武电厂“东热西送”建设，建成西夏热电二期，拆除60台20蒸吨以下燃煤小锅炉，推进秸秆收集综合利用，新购100辆新能源公交车，更换一批新能源出租车，空气质量优良天数达到76%；扩整提升6760亩湖泊湿地，启动滨河生态动物园建设，完善贺兰山东麓防洪体系及葡萄产业带水利配套设施建设。升级改造一二四五污水处理厂，达到一级A排放标准。

（四）抓好改革与开放“两大任务”。坚持先行先试，为国家、自治区试水探路。深化供给侧结构性改革。落实国家支持实体经济各项政策，提升服务供给水平，推动向中高端转型发展。去产能，稳妥做好“僵尸企业”破产清算、下岗职工分流安置；淘汰化工、冶金等落后产能6万吨。去库存，研究出台释放城镇居民改善性住房需求、农民工和农民市民化购房配套政策，规范发展住房租赁市场，消化库存600万平米。去杠杆，支持商业银行不良贷款证券化、股权化处置，依法打击非法集资犯罪。降成本，落实营改增和区市工业扶持政策，低成本化改造工业园区2家，争取电力直接交易减少企业成本9000万元，全面清理规范政府性基金和收费，再取消一批行政事业性收费项目；补短板，加大力度补好基础设施、公共服务、人力资源、生态环境等领域短板。深化“放管服”改革。再取消和下放一批行政职权。推进电子证照、电子签章应用和“一窗申请、一表登记”，同步启动多证联办等商事制度改革。加快完善市县乡一体架构、多级联动体系，力争网上审批达到60%以上。搭建审批监管互动、咨询投诉、综合执法信息平台，强化事中、事后监管。开展“减证便民”专项行动，最大程度方便群众和企业。深化金融创新。做大做强银川金融资产交易中心，打造全国PE之城；支持企业开展离岸金融、海外债发行等创新，大力发展金融科技。新增“新三板”挂牌企业10家。建设市综合治税管理信息平台，增强国、地税联合办税服务。深化农村综合改革。全面启动“两证合一”确权颁证，稳妥推进农村土地“三权分置”，健全农村集体“三资”管理监督和收益分配制度，增加农民财产性收入，发展新型农村合作金融。深化国资国企改革。完善以管资本为主的监管体系，强化目标考核，推进国企高管去行政化，建立现代职业经理人制度。支持国有企业开展战略合作或兼并重组，发展混合所有制经济。做强做优开放平台。滨河新区实施项目78个，力争完成投资160亿元。综保区引进拓展国际交易结算、综合信息服务、设备租赁、检测咨询等业务，力争实现进出口总额30亿元以上。阅海湾商务区加快“全球汇”二期、创天国际媒体产业中心等项目建设，力争完成投资120亿元。提升通道开放水平。陆路，推进共建乌力吉口岸，启动实施机场高速专线、古青高速北延伸工程，打通与鄂尔多斯、榆林等周边区域路网。空中，完成国际航空港综合交通枢纽工程，开辟更多国际客货运航线；加快临空经济区建设，推进综保区与河东机场航空港融合发展。网上，大力培育和引进丝路通等跨境电商企业，建设跨境电商交易平台和第三方综合服务平台，完善通关、结汇、退税等机制，拓展多层次网上国际贸易。

（五）实施民生“七大计划”。民生无小事，枝叶总关情。重点办好20件民生实事，持续保障和改善民生，增加人民群众获得感。实施精准脱贫计划。深化推广闽宁协作模式，抓好闽宁产业城和闽宁扶贫产业园等东西扶贫协作项目。全面落实各项精准脱贫政策措施，推进脱贫攻坚与农村低保制度有效衔接。完成10081名移民搬迁安置任务，力争13个移民村脱贫，1.4万贫困人口脱贫销号，移民人均收入增长11%。实施创业增收计划。抓好高校毕业生就业创业，建成一批低成本创业孵化基地、创客空间，健全完善下岗职工、残疾人、农民工等困难群体终身职业技能培训制度，零就业家庭实现动态清零。城镇新增就业4万人，农村劳动力转移就业8万人。实施社会保障提标计划。推进智慧社保“诊疗一卡通”。提高城乡居民基础养老金和医保补助标准，接轨全国城乡居民异地就医直接结算。全面推进国家医养结合试点城市建设，出台养老服务规范性制度。提高城乡低保标准和救助水平。推动社会慈善体系建设，争创全国七星级慈善城市。实施教育提质计划。深化政府购买普惠性幼儿园教育服务，抓好横城等10个学前教育项目。实施北京师范大学附属学校、北塔中学迁建等义务教育项目30个,信息化改造中小学100所。实行农村籍普通高中生免费教育。实施宁夏幼儿师范高等专科学校二期、兰州大学银川校区项目，推动校企合作、产教融合。实施健康银川计划。加快公立医院综合改革，全面推行分级诊疗，打造医疗服务联合体，推行家庭医生签约服务。动态调整按病种分值结算体系，实行基本医疗保险、大病保险和医疗救助在医院的一站式结算。加快生命谷产业园、国际医疗城、阅海妇产分院、滨河新区综合医院等项目建设，稳妥推进“好大夫”“微医”等互联网医疗产业发展。启动“健康细胞”工程，建成上海路、镇苏路等6条慢行系统，教育引导群众形成健康的行为和生活方式，推进大健康关口前移。实施文化惠民计划。抓好银川演艺中心和30个农村综合性文化服务中心建设，推进“四送六进”“花开四季”等群众特色文化活动常态化、品牌化。加快发展文化创意、影视动漫、知识产权交易等业态，成立小微文化企业联盟，支持宁夏网虫、智慧宫等文化创意企业拓展海外业务。实施社会治理创新计划。新建80个智慧社区，加强社区协商自治，推进物业服务提档升级，利用信息技术严密防范和依法精准打击各类违法犯罪活动，提升城乡综合治理水平。开展安全生产“十大专项整治”，坚决遏制重特大安全生产事故发生。深入开展食品药品专项整治，保障市民生命健康安全。完善防灾减灾、应急处置体制机制，提升灾害防御能力。启动全国民族团结模范市创建工作。推进军地融合，争取全国国防教育动员现场会在银召开。

四、加强政府自身建设，提高施政能力和服务水平

打铁还需自身硬，重任千钧惟担当。新一届政府要把全面建成小康社会的使命扛在肩上，把万家忧乐放在心头，努力建设人民满意的法治政府、创新政府、廉洁政府和服务型政府。

第一，严格依法行政。认真落实国家法治政府建设实施纲要，严守政治规矩、把握市场规律、遵循法治规则，自觉接受人大法律监督、工作监督和政协的民主监督，主动接受社会监督，认真做好人大议案建议和政协提案办理工作。严格执行重大事项请示报告、政府议事规则等各项制度规定，把政府活动全面纳入法治轨道。推进“七五普法”。政府工作人员要带头学法、守法、用法，自觉运用法治思维和法治方式推动工作，切实做到法无授权不可为，法定职责必须为。

第二，担当克难实干。完善绩效考评体系，重大任务重点工作全部建立台账，实行领导包抓、部门包干和责任人包定制度，责任不落实不放过，任务不完成不放过。完善行政过错追究、干部容错纠错机制，定的就坚决干、尽快干，错的就坚决改、尽快改。建立突出问题整改销号制，下决心解决一批事关群众切身利益和久拖不决的难题。坚持以人民为中心，对照群众期盼看一看担当克难实干劲头足不足，面对矛盾敢于迎难而上，面对危险敢于挺身而出，面对失误敢于承担责任，团结带领干部群众干出一番事业。

第三，优化政务服务。综合运用市场化、社会化、信息化等手段，继续深化政务改革，优化发展环境。推进市县乡三级政务服务系统互联互通，实现信息资源共享和业务协同办理。扩大政府购买公共服务领域和范围，凡适合由社会组织提供的公共服务和解决的事项，原则以政府采购方式向社会购买；凡属于事务性公共服务，原则上一律引入市场竞争机制。探索建立行业协会监管、第三方评估考核、行政综合服务机制。拓展提升“12345”便民政务服务平台功能。深入推进政务公开，运用传统媒体和网络等新兴媒体，及时传递政务信息、回应社会关切，拉近政府与群众距离。

第四，加强廉洁自律。深入贯彻中央“八项规定”精神和区市若干规定，巩固“三严三实”“两学一做”教育成果，把全面从严治党贯穿工作始终。严格落实“两个责任”，履行“一岗双责”，强化《准则》和《条例》等纪律约束，建立健全廉政和业务工作融合机制，严格执行政府权力清单、责任清单和市场准入负面清单，减少权力寻租空间、铲除腐败滋生土壤。加强行政监察，强化审计监督，严肃执纪问责，持续加大“三不”案件查办力度，零容忍惩治腐败。努力锻造一支恪尽职守、夙夜在公、敢于负责、勤政廉洁的干部队伍。

各位代表，同志们，梦想孕育奇迹，万事出自艰辛。创新发展、转型追赶的重任落在了我们肩上，历史的接力棒交到了我们手中，政府责无旁贷。在新的长征路上，我们要更加紧密地团结在以习近平同志为核心的党中央周围，在自治区党委、政府和市委的坚强领导下，凝聚起全市各族人民的智慧和力量，不忘初心，继续前进，以铁一般的信仰，铁一般的信念，铁一般的纪律，铁一般的担当，为建设“两宜两化”和西北重要中心城市而努力奋斗！以优异的成绩迎接党的十九大和自治区第十二次党代会胜利召开！
